# The aldose reductase inhibitors AT-001, AT-003 and AT-007 attenuate human keratinocyte senescence

**DOI:** 10.3389/fragi.2024.1466281

**Published:** 2024-12-17

**Authors:** Gautham Yepuri, Kushie Kancharla, Riccardo Perfetti, Shoshana Shendelman, Andrew Wasmuth, Ravichandran Ramasamy

**Affiliations:** ^1^ Diabetes Research Program, Holman Division of Endocrinology, Diabetes and Metabolism, Department of Medicine, New York University School of Medicine, New York, NY, United States; ^2^ Applied Therapeutics Inc, New York, NY, United States

**Keywords:** aldose reductase, aldose reductase inhibitors, skin cell aging, senescence, oxidative stress

## Abstract

Human skin plays an important role protecting the body from both extrinsic and intrinsic factors. Skin aging at cellular level, which is a consequence of accumulation of irreparable senescent keratinocytes is associated with chronological aging. However, cell senescence may occur independent of chronological aging and it may be accelerated by various pathological conditions. Recent studies have shown that oxidative stress driven keratinocyte senescence is linked to the rate limiting polyol pathway enzyme aldose reductase (AR). Here we investigated the role of three novel synthetic AR inhibitors (ARIs) AT-001, AT-003 and AT-007 in attenuating induced skin cell senescence, in primary normal human keratinocytes (NHK cells), using three different senescence inducing agents: high glucose (HG), hydrogen peroxide (H_2_O_2_) and mitomycin-c (MMC). To understand the efficacy of ARIs in reducing senescence, we have assessed markers of senescence, including SA-β-galactosidase activity, γ-H2AX foci, gene expression of *CDKN1A, TP53* and *SERPINE1*, reactive oxygen species generation and senescence associated secretory phenotypes (SASP). Strikingly, all three ARIs significantly inhibited the assessed senescent markers, after senescence induction. Our data confirms the potential role of ARIs in reducing NHK cell senescence and paves the way for preclinical and clinical testing of these ARIs in attenuating cell aging and aging associated diseases.

## Introduction

Skin aging is a complex process that is governed by key contributions from several factors (Farage et al., 2008). Gender, ethnicity, genetic variations, inherited genes, radiation exposure, diabetes, nutrition, environment and lifestyle habits contribute to the process of skin aging (Farage et al., 2017; Gunn et al., 2014; Makrantonaki et al., 2012; Green et al., 2011; Tobin, 2017; McDaniel et al., 2018; Argyropoulos et al., 2016; Panich et al., 2016). The process of aging is initiated at cellular level by loss of function and ability to replenish damaged keratinocytes. This process, defined as senescence of keratinocytes, has an overall adverse impact on skin aging. The hallmark of cellular senescence is associated with increase of both cytosolic and mitochondrial reactive oxygen species production (ROS), expression of key senescence markers including SERPINE1/PAI1, cell cycle inhibitors, such as CDKN1A/p21, pro-apoptotic marker genes, including TP53/p53 and release of senescence associated secretory phenotype (SASP) (Vaughan et al., 2017; Yepuri et al., 2016). Furthermore, Histone γ-H2AX foci, sensitive marker of double-stranded DNA breaks (DSBs) and telomere shortening increases in damaged and senescent cells (Hovest et al., 2006; Sedelnikova et al., 2004; Kaul et al., 2011).

Impaired carbohydrate metabolism plays a key role in the pathogenesis of obesity, diabetes, and metabolic syndrome, and consequently associated with skin diseases (Stefanadi et al., 2018; Ness, 2004; Lakdawala et al., 2013; Cosgrove et al., 2007; Danby, 2010). Several studies (Jannapureddy et al., 2021; Vedantham et al., 2012; Ramasamy and Goldberg, 2010) have demonstrated that increase in aldose reductase (AR) enzymatic activity is associated with oxidative stress, and AR inhibition reduces ROS and advanced glycation end products, which has been linked to skin cell senescence (Jannapureddy et al., 2021; Vedantham et al., 2012; Ramasamy and Goldberg, 2010; Ananthakrishnan et al., 2009). Furthermore, in the context of skin cells, Farres and colleagues (Ruiz et al., 2011; Ruiz et al., 2009) established that AR is able to catalyze reduction of retinal to retinol *in vitro*. A previous study (Yepuri et al., 2016; Kang et al., 2011) demonstrated that normal human keratinocytes express AR strongly compared to other enzymes proposed to be physiologically important retinal reductases. Recently, we demonstrated the link between AR activity and senescence in subcutaneous adipose tissue of mice fed with high fat diet (Thiagarajan et al., 2022). Here, we propose that AR is a potential therapeutic target for beneficial modulation of skin cell properties and that reducing AR activity in human keratinocytes is a potential mechanism to modify keratinocyte senescence. In our present study we tested the role of potent and selective AR inhibitors (ARIs) AT-001, AT-003, AT-007 (Mylari, 2014a; Mylari, 2014b; Shendelman, 2018; Wasmuth et al., 2013; Wasmuth et al., 2014), in progression of AR induced human keratinocyte senescence.

## Methods

### Cell culture and treatment

Normal pooled adult Human Epidermal Keratinocytes (NHK) were obtained from PromoCell (cat # C-12006) maintained and passaged in serum free Keratinocyte complete growth medium (PromoCell, cat # C-20111). Early passage cells (P4-P6) were used for all our studies. To induce senescence and asses the effect of AT compounds on senescence phenotype we treated cells either with 200 nM Mitomycin (cat # 10107409001) for 24 h, 25 mM of D-glucose to mimic high glucose conditions and 10 µM of H_2_O_2_ for 48 h or 400 µM for 1 h followed by washout and consequently cells were analyzed for senescence markers at day 10. In treatment groups, AT compounds (AT-001, AT-003, AT-007) were added at a concentration of 0.2 nM 10 min prior to senescence induction. For all compounds, 1 mM stock solutions were prepared in DMSO, consequent dilutions to 0.2 µM stocks were prepared in PBS under sterile conditions. Vehicle also referred to as Veh which was added to control cells were prepared accordingly. The dose used is calculated based on EC50 for all AT compounds (Shendelman, 2018; Wasmuth et al., 2013; Wasmuth et al., 2014).

### Human AR activity assay

Colorimetric assessment of human AR activity was performed using AR Activity Kit (BioVision, Inc. cat # K369) as per manufacturer’s instructions. Absorbance was measured at 340 nm for a duration of 60 min at a regular time interval of 90 s using TECAN Infinity Pro 200 plate reader. Calculations were performed as per manufacturer’s instructions. The resultant data was normalized to total protein, which was assessed using Pierce™ BCA Protein Assay Kit (Thermo Scientific™ cat # 23225).

### Senescence associated **β** galactosidase staining (SA-**β**-gal)

NHK cells were plated in standard six well plates. SA-β-gal staining was performed using Cellular Senescence Staining Kit obtained from Cell Biolabs, Inc. (cat# CBA230) as per manufacturer guidelines. In brief, cells were washed and fixed by the fixative provided by the kit. Cells were incubated with SA-β-gal staining solution for 16 h. Cells were counter stained with Syto-13 (ThermoFisher Scientific cat# S7575) dye to obtain accurate cell count. Alternatively, florescent based approach was used to measure cellular senescence using CellEvent™ Senescence Green Detection Kit from ThermoFisher Scientific (Cat# C10851). The experiment was performed as per manufacturer’s guidelines. Cells were counter stained with DAPI to obtain accurate cell count. Images were taken at ×10 magnification using Ziess Axio-observer light and florescent microscope. For CellEvent™ Senescence Green Detection Kit, images were taking at 488 nm filter set. Cell count was performed using NIH ImageJ software.

### Cytosolic and mitochondrial superoxide measurements

NHK cells were plated in 12 wells plates. Post respective treatments, cells were stained with live cell superoxide indicators. For detecting cytosolic superoxide, cells were incubated with 5 µM dihydroethidium (ThermoFisher Scientific Inc. cat # D1168) for 30 min. To detect mitochondrial superoxide, cells were incubated with 5 µM MitoSOX dye (ThermoFisher Scientific, cat# M36008) for 10 min. Cells were later washed with PBS to remove excess dye and fixed with 3.7% paraformaldehyde (PFA). Imaging was performed at ×10 magnification using EVOS XL Core Imaging System. Relative intensity for images was calculated using NIH ImageJ software.

### Quantitative PCR

RNA from treated cells was extracted using RNeasy Plus Mini Kit (QIAGEN, cat#74134) and cDNA was synthesized using qScript™ cDNA SuperMix (Quanta BioSciences, Inc. cat # 95048). RT-qPCR was performed using Taqman gene expression assays. 20 ng of RNA input per reaction was used for all qPCR studies. Amplification reactions were performed by Applied Biosystems 7500 Fast Real-Time PCR System. Relative fold change in expression for desired genes was performed using the ΔCT method of analysis. All gene expression data were normalized to housekeeping gene *PPIA*. Genes used to determine expression are listed in Table 1.

**TABLE 1:** Human Taqman qPCR primers.

Taqman catalog #	Human gene
Hs00355782_m1	*CDKN1A*
Hs01034249_m1	*TP53*
Hs00167155_m1	*SERPINE*
Hs00923894_m1	*CDKN2A*
Hs01059205_m1	*LMNB1*
Hs04194521_s1	*PPIA*

### ELISA for cytokine measurements

ELISA was performed to determine cytokine release into Condition medium. Condition medium at day 10 was collected from skin cells treated H_2_O_2_ as described above. ELISA was performed for cytokines released in condition medium for IL1β (Abcam, cat # ab214025), TNFα (Abcam, cat # ab181421), MCP1 (Abcam, cat # ab179886) and IL6 (Abcam, cat # ab178013) as per manufacturers protocols. All data was normalized to total protein content assessed by BCA assay. Absorbance as described in the protocol was measured using TECAN infinity pro 200 plate reader.

### Gamma H2A.X staining

To measure DNA damage in NHK cells treated with long term H_2_O_2_, Gamma H2A.X Staining Kit (ABCAM; cat # ab242296) was used. Cells were stained according to company protocol followed by florescent imaging using EVOS XL Core Imaging System at ×10 magnification. Relative intensity and cell count for images was calculated using NIH ImageJ software.

### Statistical analysis

All data was analyzed using GraphPad PRISM 9 software (GraphPad, LaJolla, CA). n represents biological replicates obtained from two consecutive batches performed at two different time points with cells originating from separate source. Each batch comprised of n = 2 obtained from two separate culture dishes or wells. All data are expressed as mean ± SEM. Unpaired t-test was performed to compare data between two groups and Ordinary one-way ANOVA with Tukey’s multiple comparisons test was implemented for comparison between more than two groups. p < 0.05 was considered statistically significant between groups.

## Results

### AR activity is increased in senescent human keratinocytes

**FIGURE 1:**
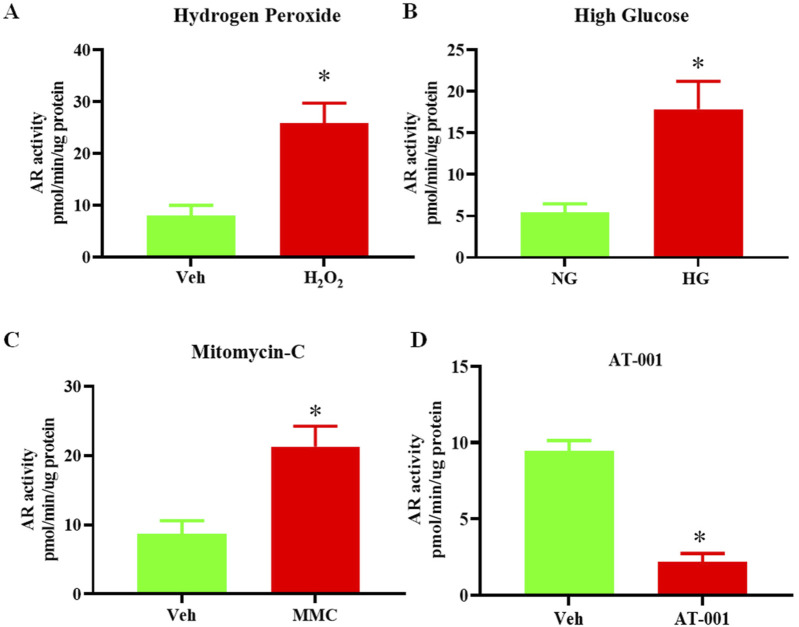
Senescence induction upregulates AR activity. AR activity measured by colorimetric assay in human keratinocytes either treated with **(A)** 10 uM H_2_O_2_ or Vehicle (Veh- dH_2_O) **(B)** 25 mM high glucose (HG) or normal glucose (5 mM) (NG) for 48 h **(C)** 200 nM Mitomycin-C (MMC) for 24 h compared to Veh (DMSO) and **(D)** 0.2 nM AT-001 for 48 h vs. Veh (1:5,000 dilution DMSO in PBS). Data are presented as the mean ± SEM. n = 4 per group obtained from two consecutive experiments. Unpaired t-test was implemented for comparison between two groups. p* < 0.05 vs. Veh or NG.

Previous studies have demonstrated the deleterious effect of the activity of the polyol pathway in accelerating keratinocyte senescence (Kang et al., 2011). To determine if senescence is linked to changes in AR activity, we induced senescence by treating human keratinocytes either with mitomycin C (MMC), H_2_O_2_ or high glucose (HG) and measured AR activity. We observed a 3-fold increase in AR activity upon treatment with 10 µM H_2_O_2_ or 25 mM D-glucose compared to controls (Figures 1A, B) and a 2.5-fold increase upon treatment with 200 nM MMC (Figure 1C). These data link increases in activity of polyol pathway enzyme AR in keratinocyte senescence.

### ARIs prevent HG induced senescence

**FIGURE 2:**
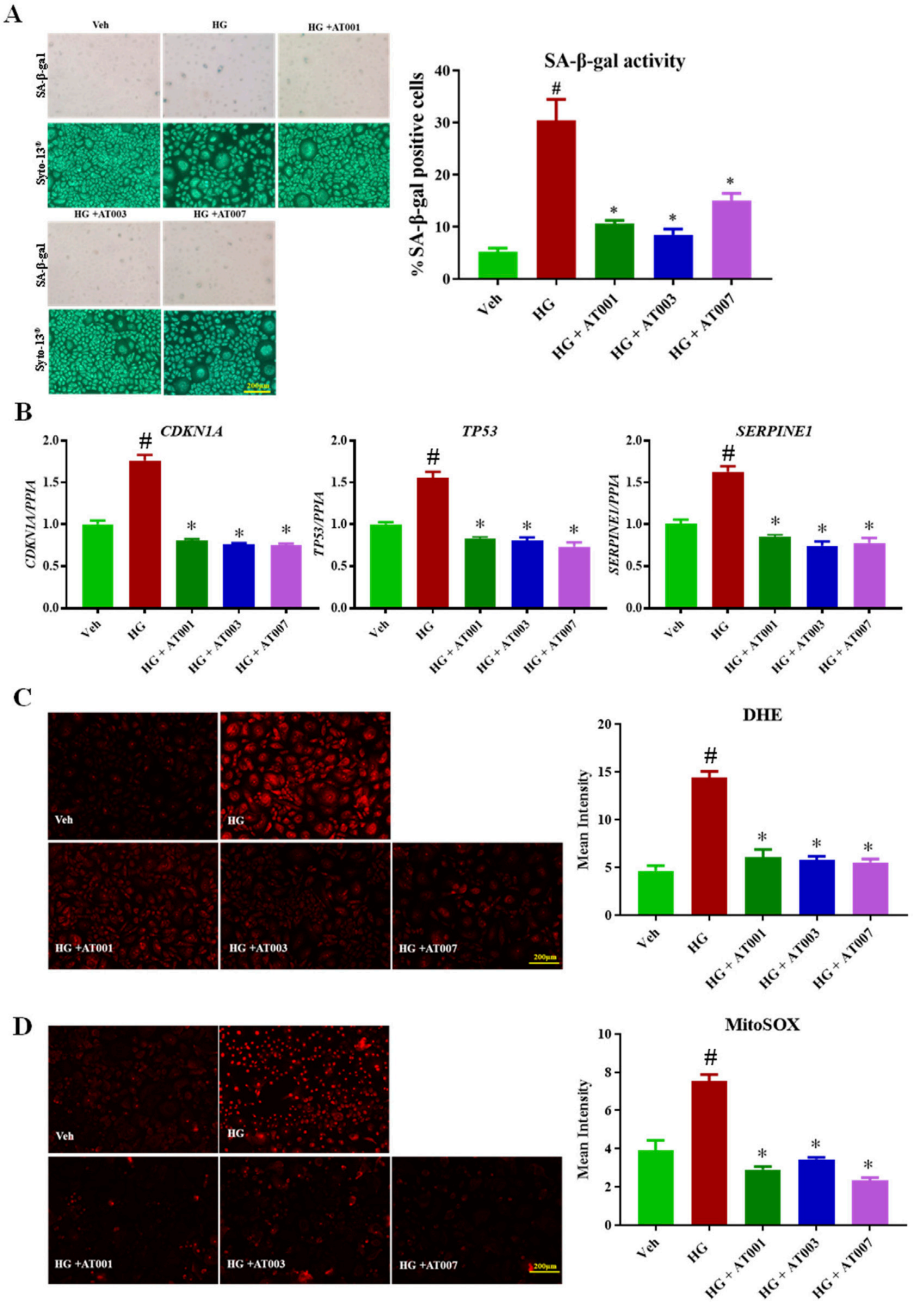
ARIs prevent HG induced senescence phenotype. Human keratinocytes were treated with 25 mM HG or normal condition medium NG. **(A)** SA-β-gal staining was performed to assess positive senescent cells. **(B)** qPCR analysis for confirming gene expression of senescent markers *CDKN1A, TP53* and *SERPINE1*. **(C)** Skin Cells stained with 5 µM DiHydroethidium (DHE) dye for 30 min to detect cytosolic superoxide and **(D)** cells stained with 5 µM MitoSOX dye for 10 min to detect mito superoxide. Data are presented as the mean ± SEM. n = 4 per group obtained from two consecutive experiments. Ordinary one-way ANOVA with Tukey’s multiple comparisons test was implemented for comparison between groups. p* < 0.05 vs. NG. p# < 0.05 vs. Veh.

The main focus of our study is to test the preventive role of three different, novel and highly-selective ARIs, AT-001, AT-003 and AT-007 in induced keratinocyte senescence. We initially tested if AT-001, which is the parent drug for AT-003 and AT-007 can inhibit AR activity in normal keratinocytes and our data clearly demonstrated more than 75% reduction in AR activity upon AT-001 treatment compared to vehicle treatment (Figure 1D). SA-β-gal staining, gene expression of *CDKN1A*, *TP53* and *SERPINE* and both cytosolic and mitochondrial (mito) ROS superoxide production are well-established gold standard markers for cellular senescence. To test our hypothesis, we measured these key markers to confirm the preventive role of ARIs on human keratinocyte senescence. As it was evident that all three senescence-inducing agents significantly increased AR activity, we next wanted to investigate if ARIs prevented HG induced senescence. Treatment with HG for 48 h induced senescence by 30% as demonstrated by SA-β-gal staining (Figure 2A). Treatment with all three compounds 10 min prior to addition of HG significantly prevented HG induced senescence, of which AT-001 and AT-003 demonstrated maximum inhibition. Morphology observed by cell tracker dye Syto-13^®^ displayed fewer flattened cells (typical morphology of senescent cells) in ARI treatment groups compared to the control vehicle-treated HG group (Figure 2A). We next confirmed significant downregulation of HG increased gene expression *CDKN1A, TP53* and *SERPINE* by all three ARIs (Figure 2B). Furthermore, HG induced cytosolic and mito superoxide generation, which was measured by DHE and MitoSOX dye respectively was also strongly inhibited by AT-001, AT-003 and AT-007 (Figures 2C, D). Our data above provides strong evidence and emphasizes the importance of these compounds in preventing HG induced cellular senescence.

### ARIs prevent hydrogen peroxide induced senescence

**FIGURE 3:**
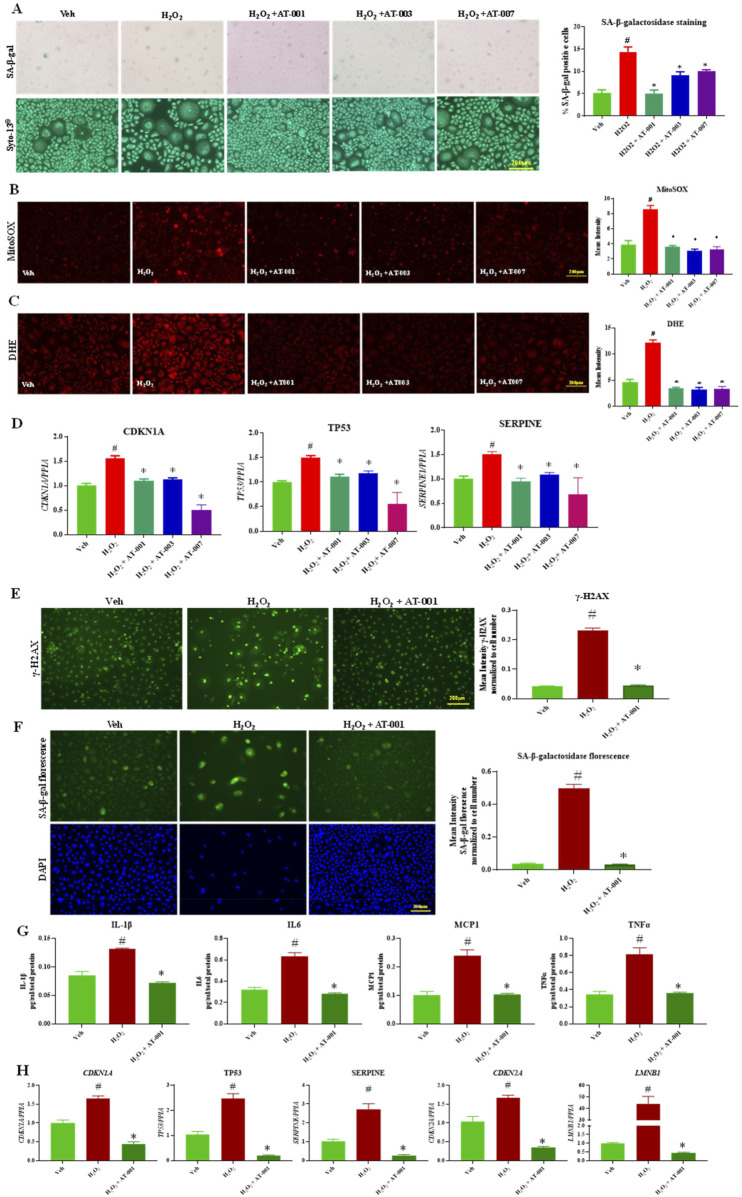
ARIs prevent H2O2 induced senescence phenotype. Human keratinocytes were treated with 10 µM H_2_O_2_ or Veh (dH_2_O) and ARIs for 48 h. **(A)** SA-β-gal staining was performed to assess positive senescent cells. **(B)** Skin Cells stained with 5 µM DiHydroethidium (DHE) dye for 30 min to detect cytosolic superoxide and **(C)** cells stained with 5 µM MitoSOX dye for 10 min to detect mito superoxide. **(D)** qPCR analysis for confirming gene expression of senescent markers *CDKN1A, TP53* and *SERPINE1*. **(E)** Gamma H2A.X Staining and **(F)** CellEvent™ Senescence Green Detection staining in human keratinocytes treated with Veh (dH_2_O), 400 µM H_2_O_2_ for 1 h followed by assessment of cells at day 10 with or without ARI AT-001. **(G)** ELISA to measure cytokine release in condition medium collected at day 10 and **(H)** qPCR analysis for confirming gene expression of senescent markers *CDKN1A, TP53* and *SERPINE1 CDKN2A and LMNB1*. Data are presented as the mean ± SEM. n = 4 per group obtained from two consecutive experiments. Ordinary one-way ANOVA with Tukey’s multiple comparisons test was implemented for comparison between groups. p* < 0.05 vs. H_2_O_2_. p# < 0.05 vs. Veh.

H_2_O_2_ is a well-known chemical agent, which causes senescence and increases reactive oxygen species generation in cells (Ramis et al., 1989). We next tested the efficacy of ARIs in preventing H_2_O_2_ induced senescence phenotype. Exposure to 10 µM H_2_O_2_ for 48 h significantly upregulated all the key markers of senescence tested with a 3-fold increase in SA-β-gal positive keratinocytes (Figure 3A). Treatment with the ARI compounds 10 min prior to addition of H_2_O_2_ significantly inhibited H_2_O_2_ induced senescence phenotype of which AT-001 completely abolished H_2_O_2_ induced SA-β-gal positive staining (Figure 3A). H_2_O_2_ induced DHE and MitoSOX staining was significantly inhibited by AT-001, AT-003 and AT-007 (Figures 3B, C). Gene expression of *CDKN1A, TP53* and *SERPINE* was consistently downregulated by all three ARIs (Figure 3D). AT-001 is the parent drug for other derivatives AT-003 and AT-007. We next wanted to investigate the efficacy of AT-001 on long term treatment of H_2_O_2._ As described in the methods, keratinocytes were initially treated with 400 µM H_2_O_2_ for 1 h with or without AT-001 and the cells were allowed to be incubated in AT-001 for 10 days. At day 10 we observed a strong increase in fluorescence signal in cells treated only with H_2_O_2_ compared to vehicle treated group for γ-H2AX stain which is a marker for DNA damage and impaired cell proliferation. The fluorescence signal was abolished in cells co-treated with AT-001 and H_2_O_2._ (Figure 3E). Alternatively, we have used florescent based SA-β-galactosidase staining approach to detect senescence in these conditions. As observed in Figure 3F, AT-001 prevented florescent senescence signal induced by long term H_2_O_2_ treatment. We then assessed the SASP release into condition medium in keratinocytes upon H_2_O_2_ treatment. We observed significantly higher release of SASP IL1β, IL6, MCP1 and TNFα in H_2_O_2_ treated cells and that treatment with AT-001 reduced the increases in SASP (Figure 3G). Long term exposure of H_2_O_2_ significantly increased gene expression of *CDKN1A, TP53, SERPINE, CDKN2A* and well-established keratinocyte senescence marker *LMNB1 (* Figure 3H), AT-001 treatment blocked the increases in all these senescent marker genes (Figure 3H).

### ARIs prevent mitomycin induced senescence

**FIGURE 4:**
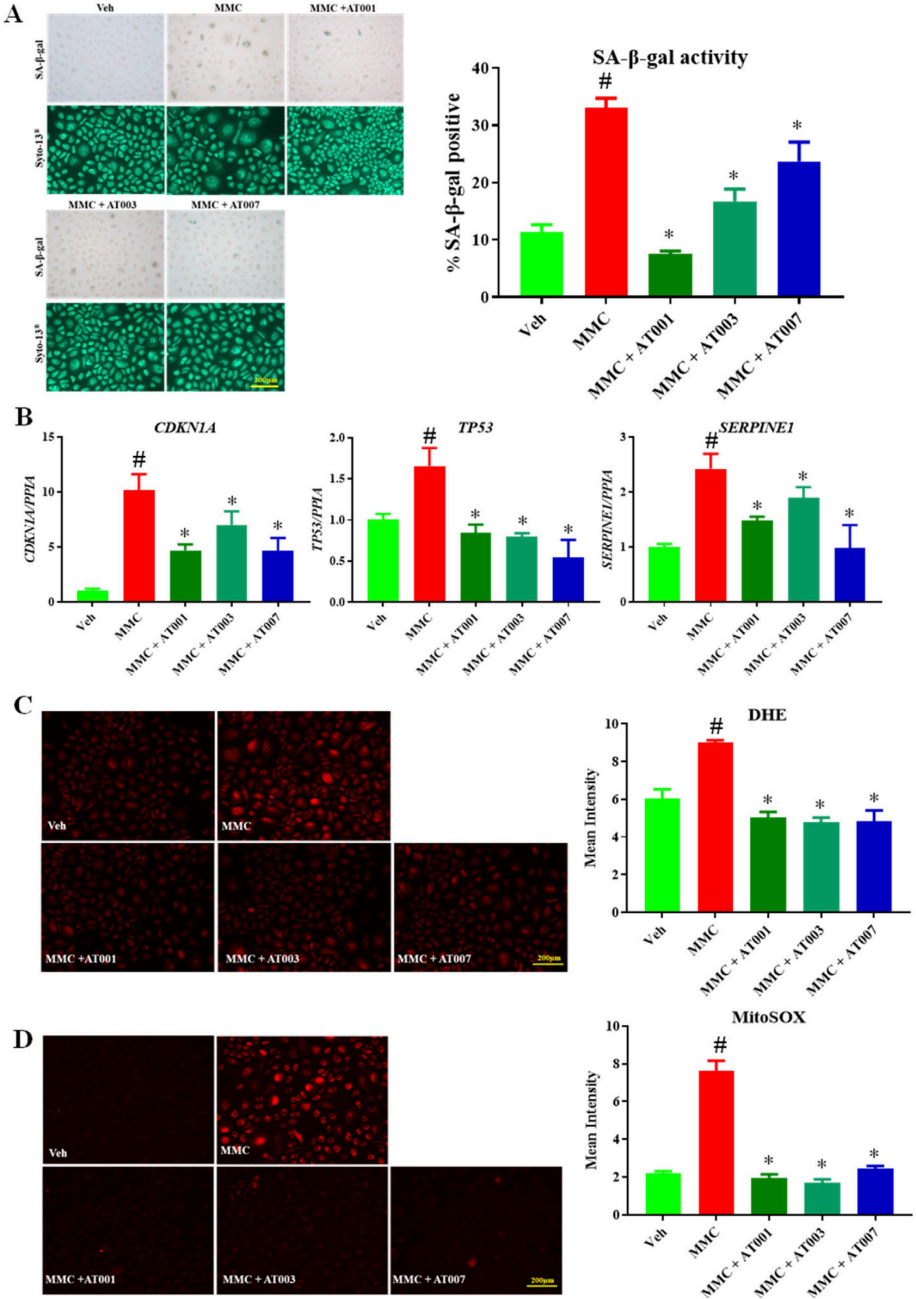
ARIs prevent MMC induced senescence phenotype. Human keratinocytes were treated with 200 nM MMC or Veh (DMSO) and ARIs for 24 h. **(A)** SA-β-gal staining was performed to assess positive senescent cells. **(B)** qPCR analysis for confirming gene expression of senescent markers *CDKN1A, TP53* and *SERPINE1*. **(C)** Skin Cells stained with 5 µM DiHydroethidium (DHE) dye for 30 min to detect cytosolic superoxide and **(D)** cells stained with 5 µM MitoSOX dye for 10 min to detect mito superoxide. Data are presented as the mean ± SEM. n = 4 per group obtained from two consecutive experiments. Ordinary one-way ANOVA with Tukey’s multiple comparisons test was implemented for comparison between groups. p* < 0.05 vs. MMC. p# < 0.05 vs. Veh.

To strengthen our hypothesis, we have used a third approach which is drug induced accelerated senescence approach. Here, we treated with 200 µM of MMC for 24 h which induced approximately 35% SA-β-gal positive keratinocytes (Figure 4A). Consistent with our above finding, all three compounds significantly prevented MMC induced senescence (Figure 4A). In addition, all three compounds significantly downregulated MMC induced gene expression of *CDKN1A, TP53* and *SERPINE*. MMC treatment induced cytosolic and mito superoxide production was consistently prevented with AT-001, AT-003 and AT-007 (Figures 4B–D). Taken together our data demonstrates that all three ARI compounds were effective in attenuating human keratinocyte senescence.

## Discussion

Induction of senescence in fibroblasts by exposure to high glucose and its link to AR expression was first demonstrated by Sibbitt et al. (1989). Our previous study in adipose tissue demonstrated an increase in gene expression of AKR1B3, the mice isoform of human AKR1B1, the gene encoding for the aldose reductase enzyme at day 7 of high fat diet feeding (Thiagarajan et al., 2022). In this study, we demonstrated that increased AR activity precedes increased AR gene expression, occurring within 48 h of high glucose or H_2_O_2_ treatment, and within 24 h of MMC treatment. This is in line with prior studies demonstrating that AR activity can be regulated post-transcriptionally, and AR activity is often independent of expression upregulation (Thiagarajan et al., 2022).

This study and earlier studies in fibroblasts and adipose tissue clearly demonstrate a link between induction of senescence and AR expression (Thiagarajan et al., 2022; Sibbitt et al., 1989). However, it should be noted that a study using DNA-damage induced senescence model AR appeared to have the opposite effect on senescence (Kang et al., 2011) than what is observed in this study. This difference in AR axis in senescence is, perhaps, attributable to the DNA-damage induced vs. H_2_O_2_ and MMC induced models of senescence.

ROS generation and oxidative stress are well established mechanisms which lead to accelerated senescence phenotype (Yepuri et al., 2016; Yepuri et al., 2012). Increased AR activity has been linked to increased ROS generation and oxidative stress in multiple cell types (Jannapureddy et al., 2021; Ananthakrishnan et al., 2009; Lee and Chung, 1999; Srivastava et al., 2011; Tang et al., 2014; Tang et al., 2008; Williamson et al., 1993). In addition, our earlier studies demonstrated that flux via AR downregulates NAD + dependent SIRT1 activity and drives hyperacetylation of the transcription factor EGR1 under hyperglycemic conditions and ischemic conditions in cardiovascular cells (Vedantham et al., 2014). It is well established in the literature that SIRT1 activity and expression are key regulators of cellular senescence (Chen et al., 2020; Hwang et al., 2013; Lee et al., 2019). It is possible that AR may mediate senescence, in part, vis SIRT1 axis. Clearly, the novel AR inhibitors, AT-001, AT-003 and AT-007, are effective in reducing oxidative stress, along with downregulation of gene expression of key senescence markers, SA-β-gal activity, and SASP. Taken together, all three compounds demonstrated high efficacy in retarding senescence in human keratinocytes.

The skin, considered as the largest organ of the human body, plays an important role in protecting the body from both intrinsic and extrinsic stressors. Additionally, skin aging has broader implications for longevity and cardiometabolic complications (Franco et al., 2022). The protective role of ARI compounds in skin cell senescence have created a new direction and opportunity for testing AR inhibitors in attenuating skin aging.

## Conclusion

**SCHEME 1:**
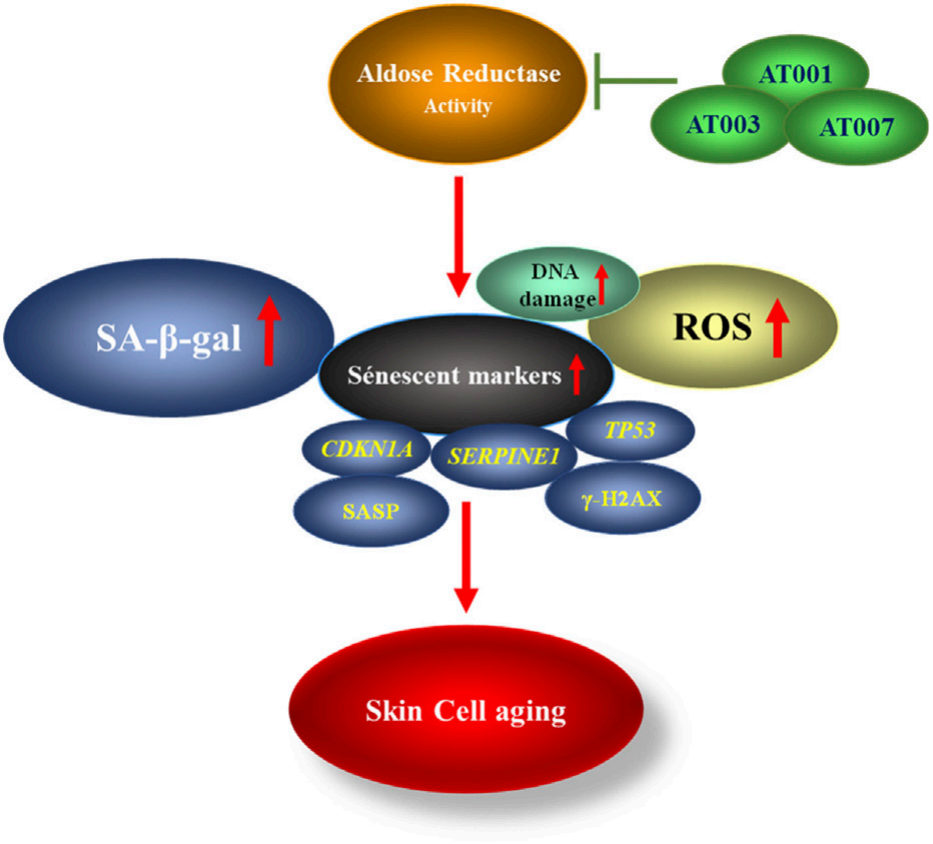
Schematic representation showing AR activity increases senescent markers thereby promoting skin cell aging which can be inhibited by treatment with ARIs.

In this study, we demonstrate that treatment of skin cells with high glucose, mitomycin-c and H_2_O_2_ induces senescence via increased AR activity (Scheme 1). Treatment of skin cells with AR inhibitors prevented oxidative stress and ROS generation and attenuated senescence markers (Scheme 1). These data indicate a potential role for potent and selective AR inhibitors, such as AT-001, AT-003 and AT-007, in attenuating skin aging.

## Data Availability

The original contributions presented in the study are included in the article/Supplementary Material, further inquiries can be directed to the corresponding author.
